# Research based instruction in the teaching of islamic education

**DOI:** 10.1186/2193-1801-3-755

**Published:** 2014-12-19

**Authors:** Abas Asyafah

**Affiliations:** Department of General Studies (MKDU), Faculty of Social Science Education (FPIPS), Indonesia University of Education (UPI), Jalan. Dr. Setiabudhi No.229, Bandung, West Java Indonesia

**Keywords:** Instruction, Islamic education, Students teaching and learning, Research based instruction

## Abstract

In an effort to produce skilled and competent graduates, Indonesia University of Education with its motto of “producing knowledgeable, educated and religious human-beings”, considers the development of required scientific attitudes, good behaviour, and strong religious values of students as an integral and important component needed to develop students’ noble character and their intellectual ability. Developing such qualities requires innovative teaching and learning techniques which are enjoyable and able to motivate students. In this paper, the author seeks to answer four(4) questions: 1. How can one plan a research-based instruction for an Islamic education seminar course? 2. What are the steps considered in research-based instruction for a seminar course? 3. How is the evaluation of research-based instruction in an Islamic education seminar course? And 4. What are the weaknesses and strength of the research based instruction model in regard to facilitating an Islamic education seminar? To establish an appropriate answer to these questions, the author has used a descriptive-qualitative approach. The data have been obtained through observation, interviews and a study of relevant documents. The findings reveal that the implementation of an instruction research-based model is appropriate and applicable in the teaching of Islamic education seminar course. The study concludes that instruction research based model has the capacity to engage students and it is able to improve their problem solving skills in daily life.

**Electronic supplementary material:**

The online version of this article (doi:10.1186/2193-1801-3-755) contains supplementary material, which is available to authorized users.

## Introduction

To anticipate the negative effects brought about by the developments in the field of Information and Communication technologies (ICT), and with the influence of globalisation, it is necessary for human beings to understand how to behave in society. In other words, people have to be aware of the values and virtues society wants as they face new life demands. The question arising is how to make people aware of their responsibility in such a rapidly changing world. According to Lickona (1992) people should be taught both academics and virtue or good character in order to live a noble life.

In Indonesia, to prevent students from the negative influence of ICT and globalisation, general education was developed comprising of religious education that aims to instill good values in students. This course was created to perform two major functions: first, to develop professionals and religious experts, and second, to develop knowledge and skills of students and teachers equally in respect to the varying religions found in Indonesia (Majid, 2004). Islamic education is regarded as a subject whose function is to develop and instill positive values in students through appropriate instruction process (in this case Islamic education seminar course). The objective of this course is to develop God fearing communities with good character and not to create professionals.

In reference to Indonesia’s higher education curriculum structure, Islamic education is a compulsory subject to all Muslim students in public Universities across the country. The course is considered important in national development. With the passing of the education Bill No.20/2003 into a law, the position of Islamic education became clear with its main mission of developing students’ personality. However, though Islamic education is a fundamental course within Indonesia’s National Education System, its application is still limited to conceptualisation and not yet in practice (Nasution, 1999). This is assumed to be one of the causes for moral decay in the country.

In relation to the above, this course has not been able to meet the expectations of the public. It is contrary to the four pillars of UNESCO which are learning to know, learning to do, learning to be and learning to live together. Taking the said four pillars in consideration, education should equip each individual with skills required for learning to occur (Delors et al. 1996). Using seminar as an approach to teaching and learning during lectures, there are several alternatives to be explored by both students and academics which can impact of the learning process positively. Tafsir (2006) refers to a seminar course in Islamic education as an appropriate approach and quick way to teaching and learning. A seminar as a way to instruction stimulates exchange of ideas and discussion among students. It can be powerful, if it is based on research. However, for all this entire period, this has been the weakness of teaching Islamic education. It is upon this background that the author examines research based instruction in teaching Islamic education as a seminar course.

### Literature review

The aim of teaching Islamic education is to develop and promote moral character. Studies in Islamic education have established that teaching good behaviour is an important component which enhances the development of individual potential in a holistic, balanced and integrated manner, encompassing the intellectual, spiritual, and physical aspects (Halim, 2007). Reaching the level of moral and good personality requires a process. This process can be facilitated through teaching and learning. Learning takes place only if the instructor considers students as partners, and is ready to build on what they already know and what they think (Ambrose et al., 2010). To help students understand religious values will depend on the method of instruction. If the method of instruction is not appropriate, it becomes difficult to achieve the stated education objectives. Azra (2008) points out that education aims at holistic human development. Education should therefore help people develop spiritually, intellectually, and socially. Al-Syaibani (1979) notes that there are three objectives of Islamic education: first, human objectives which are related to self improvement in form of knowledge, behaviour, intelligence and self-actualization. Second, is the social objective related to living together, and third, professional objective which takes education and learning as an important component considering Islamic education as field of knowledge, an art, and as professional as well as a social activity in the community.

Research based instruction or teaching has been developed based on three research areas, they are: research in cognitive science, research on master teachers, and research on cognitive supports. Though these are three different fields of research, they work to supplement and complement each other (Rosenshine, 2012). These three areas have led to 17 principles in regard to teaching using research based instruction as an approach to learning. Several studies have continued to show teaching and learning requires innovation. Abdulhak (2000) and Asyafah (2014) are of the view that innovative teaching and learning method must: motivate students, stimulate their desire to learn, deliver the message in a clear way, create a conducive learning environment, facilitate creativity, promote self-assessment during learning, and also promote problem-solving in learning. However, they also believe that this can work, if five points are considered before the teaching and learning process starts, that is: learning objectives, learning materials, human resource, time, and facility (Abdulhak: 2000; Asyafah, 2014). According to Tafsir (1996) and Asyafah (2014) there are several steps that can be taken during the instruction process. These steps include the Gleser model which also consists of: instructional objectives, entry-exit process, instructional steps, and assessment. Tafsir (2004) and Asyafah (2014) refer to these steps as basic concepts to knowing, doing, and being.

In an effort to establish a research based Islamic education seminar instruction model, there is need to understand the principles of instruction. According to Rosenshine (2012) there are 17 principles of Instruction, which include: staring a lesson with a short review of the previously learnt material; Presentation of new material in a gradual process while actively engaging students; Do not over load them, but introduce material in bits; Be clear and detailed during the instruction process and in explanation; use question-feedback approach to check for understanding; motivate and encourage student participation; Do more of facilitation than teaching or instructing; encourage thinking loud (letting students to talk their voice; Give example model of worked-out problems; Ask for feedback from students on what they have learned; rectify student response where necessary; Encourage being independent and autonomous; and Monitor students activities during individual practices. These are the principles established by Rosenshine. Besides this, Ihsan and Ihsan (1999) and Asyafah (2014) have also proposed nine principles of instruction, such principles include: happiness (al-Baqarah [2]: 25, 175, 155); delivery and respect (Al-Imran [3]: 159); meaning to learners ((Muhammad [47]: 16); requirement (al-Baqarah [2]:1–2, Maryam [19]: 1–2); communication (al-Araf [7]: 179, Al-Isra [17]: 37); acquiring new knowledge (al-Baqarah [2]: 164); being of good character (al-Azhab [33]: 21), (h); pushing for experience (al-Shaf [61]: 2–3); love and providing guidance (al-Anbiya [21]: 107). In reference to these principles it is necessary that whatever it is taught must be understood if people are operate in good faith and with knowledge.

Research based instruction promotes acquiring skills, strategies and behaviors (Schunk, 2012) as the most important point for learning. According to Asyafah (2014) learning is the basis for knowledge development, and also the foundation of faith and belief. It is an important activity in human life because people are the vicegerents on earth (al-Baqarah [2]: 30). Faith, belief and knowledge are important ingredients of life. A combination of knowledge, faith and belief are the basis for living a better life. A research based approach in the teaching of Islamic education must comprise “materials to be taught, presentation of materials in short steps, opportunity for learner practice, provision of collective feedback and scheduling of review sessions” (Rosenshine and Stevens, 1986; Shuell, 1988, 1990; Schunk, 2012). It is through such an instructional approach that today’s challenges facing the modern world can be avoided and or solved (Nasoetion, 2007). Combining research in the teaching and learning process through a seminar course has all the above mentioned aspects. The approach combines both theory and practice in Islamic education. To implement these two basic tasks, human beings must have a strong foundation of faith and devotion as well as adequate knowledge.

Experience without theory in most cases becomes wasteful and destructive. Having experience but without a clear guiding framework is almost nothing since one tends to treat each situation unique, hence influencing decision making which is mostly based on trial and error (Schunk, 2012). People should keep learning as knowledge is constantly changing. Learning to know presupposes to learn (Delors et al., 1996). Research based instruction which is both (scientific and philosophical) is important for the strong foundation and continuous development of the body of knowledge plus technology and also the culture of the society. It is through this approach that Islamic education’s objectives can be achieved. Islamic education as a University course aims to provide guidance and counseling to students such that they develop faith and devotion to their Creator. Nasution (1999) mentions that Islamic education in schools aims to develop student into God fearing people who able to perform all the religious rituals, while for University students, the course aims to create knowledgeable people intellectually and in personality. In Universities, Islamic education aims to: create an understanding about the teaching and learning of Islamic education.

**Figure 1:**
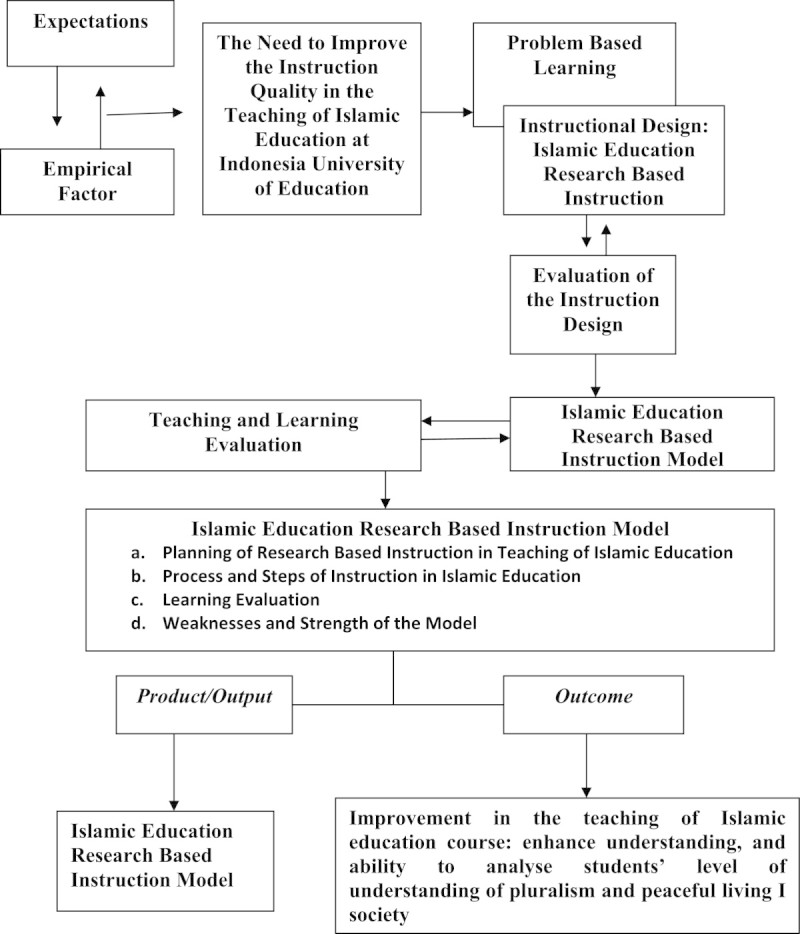
**Conceptual framework of the islamic education research based instruction.**

Teaching Islamic education requires a clear strategy which combines the method of teaching and learning theory with practice, and also the method of grouping the learning activities. The Figure 1 below illustrates this:

The figure above is a conceptual summary, illustrating the process of research as well as the conceptual framework of the study. It shows that in the process of research based instruction there is planning, instruction process, evaluation and assessing the weaknesses of the instruction model. It is designed by the author in regard to the literature studied and also based on individual interpretation.

### Research methodology

The study was conducted at the Indonesia University of Education for a period of eight (8) months from 2012 to 2013. The research was conducted in the selected classes at the University. The study comprised of planning of the study, preliminary study, field study, and writing of the research report. Since it was a qualitative study and for efficiency, the subjects of study were three (3) lecturers teaching Islamic education drawn from biology, chemistry and sociology departments. The criteria for selecting the subjects were: All subjects must be lecturers of Islamic education, they must had used the method or research in teach Islamic education for at least a year, and the selected lecturer must have taught Islamic education in both first and second semesters, that is, in the Academic year 2011/2012 and academic year 2012/2013.

They subject of study were then observed and interviewed on how they plan, the process of teaching, evaluation and assess the teaching and learning based on research. Then the weaknesses were noted throughout the whole period of the study and students were engaged to establish the strength, benefits and challenges of the research based approach during the learning process. On the side of students, 184 students were purposively chosen from three faculties: Faculty of Mathematics and Science (44 students), Faculty of Social Sciences Education (55 students) and Faculty of Languages and Art (85 students).

The data collected was analysed using descriptive qualitative method. The description meant to explain the whole process of instruction through the use of a research based model. The qualitative method was meant to help make it clear that the researchers had wanted to establish the real condition based on observation and direct interact with the subjects of the study rather than using statistical measurements. The analysis comprised of both content and primary data analysis.

### Findings and discussion

#### Planning teaching and learning process (Instruction)

This research aimed to find-out and also reveal a descriptive view about the research based instruction model in the teaching Islamic education seminar at the Indonesia University of Education. Among the aspect of research was the planning process. It was revealed that the planning process consisted of five steps, which included: modification of the syllabus, modification the course units, establishing and determining the time framework, designed student assignments and learning activities and preparation of other support equipments and materials. Schunk (2012) noted that teaching and learning emphasize various factors as important elements in acquiring skills, strategies and behaviors, while Abdulhak (2000) states that before the instruction takes place, there is a process starting from: setting learning objectives, preparing learning materials, recruiting human resource, determining the time, and also facility. Tafsir (2004) and Asyafah (2014) mentions that there are several steps that can be taken during the instruction process; among such steps are instructional objectives, entering behavior, instructional procedure, and performance Assessment. This implies that the planning is an important component before the teaching and learning process.

Preparing a syllabus is a major activity during the planning of teaching process. A syllabus works as an overview about a given subject. To teach Islamic education, there are seven points to note while developing a syllabus: course identification, objectives of the course, content description or outline, determining the learning process, evaluation procedure, breaking down the course materials and providing references. Teaching is can be categorised into two: the method of teaching and learning theory and practice, and also the method of grouping the learning activities. It means that if learning is to take place a syllabus should be developed.

A course unit is a small element in the syllabus or groupings of the course content based on the objectives. It is more detailed and aims to serve students at each meeting. It is comprised of the learning content and learning materials during each meeting. Course units in Islamic education seminar course are different from those of courses in regard to content method of delivery.

### The instruction process

The instruction process in a research based course is easy if the lecturer is able to follow the initial plan. According to researchers’ observation and the interview results, below are the steps in teaching Islamic education seminar course.

### Creating similar perception

The first step is awareness creation at first time. At this level, the lecturers described some terminologies in regard to the course content such that students are able to have almost the same understanding and perception. The explanation involved: assignments and assessment instruments. The point derived from this initial explanation was meant to inform students that process of learning and evaluation is the same.

#### Group formation

Groups were formed randomly with the help of lecturer during the first meeting. Each group consists of 9 members. After the group formation they are then arranged in order. After the group is formed the lecturer explains the tasks assigned to each group. The material is designed in a research project and students are expected to present their projects within a stated period of time. The semester ends with group discussions and presentations in form of a workshop and seminar. The format for the research based course is illustrated as in Table 1 below.

**Table 1:** **Format for a research based course**

Number	Topic or title	Background	Research problem
1.	Each group is given chance to choose a topic of its own interest but in regard to Islamic education	Writing the background setting the foundation for the study	Briefly describe the points to be raised in the research and the topic.
2.	Student creativity	Student Creativity	Creativity but not fiction

## Conclusion

The implementation of the research based method in the teaching of Islamic education has been proven appropriate both in concept and in practice. The results of this study indicate that the entering behavior is something that is very important to consider, especially in regard to ability and habit of learning Islam as well as the level of student mastery of the course. Performance assessment is very important as an effective evaluation tool in learning. The results of this study also revealed that a research based method can be applied practically in learning Islamic education to increase faith and devotion of students. This method can also be used to improve the quality of graduates.

## Authors’ original submitted files for images

Below are the links to the authors’ original submitted files for images. Authors’ original file for figure 1 Authors’ original file for figure 2
