# Supplementary figures and images for: Research based instruction in the teaching of islamic education
Source: Springerplus. 2014 Dec 19;3:755. doi: 10.1186/2193-1801-3-755 (PMC4320170; doi:10.1186/2193-1801-3-755)

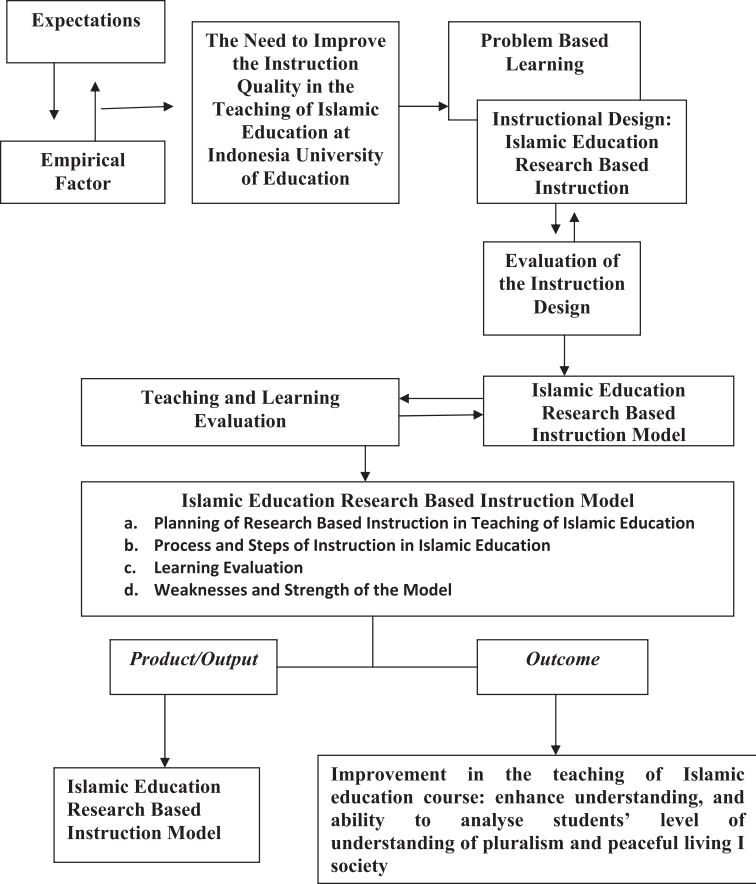

Supplement: Supplementary file 1 — Authors’ original file for figure 1 [file 40064_2014_1503_MOESM1_ESM.pdf]
