# Supplementary material for: Research based instruction in the teaching of islamic education
Source: Springerplus. 2014 Dec 19;3:755. doi: 10.1186/2193-1801-3-755 (PMC4320170; doi:10.1186/2193-1801-3-755)
Supplement: Supplementary file 2 — Authors’ original file for figure 2 [file 40064_2014_1503_MOESM2_ESM.doc]

**Figure 1. Conceptual Framework of the Islamic Education Research Based Instruction**

**Expectations**

**Empirical Factor**

**The Need to Improve the Instruction Quality in the Teaching of Islamic Education at Indonesia University of Education**

**Problem Based Learning**

**Instructional Design: Islamic Education Research Based Instruction**

**Teaching and Learning Evaluation**

**Evaluation of the Instruction Design**

**Islamic Education Research Based Instruction Model**

**Islamic Education Research Based Instruction Model**

**Planning of Research Based Instruction in Teaching of Islamic Education**

**Process and Steps of Instruction in Islamic Education**

**Learning Evaluation**

**Weaknesses and Strength of the Model**

***Product/Output***

***Outcome***

**Islamic Education Research Based Instruction Model**

**Improvement in the teaching of Islamic education course: enhance understanding, and ability to analyse students’ level of understanding of pluralism and peaceful living I society**

The format for the research based course is illustrated as in table 1 below:

| Number | Topic or Title | Background | Research Problem |
| --- | --- | --- | --- |
| 1. | Each group is given chance to choose a topic of its own interest but in regard to Islamic education | Writing the background setting the foundation for the study | Briefly describe the points to be raised in the research and the topic. |
| 2. | Student creativity | Student Creativity | Creativity but not fiction |
